# The Surgical Treatment of Retro-Deviations of the Uterus

**Published:** 1896-04

**Authors:** Augustin H. Goelet

**Affiliations:** New York


### THE SURGICAL TREATMENT OF RETRO-DEVIATIONS OF THE UTERUS.*

By AUGUSTIN H. GOELET, M.D.,

NEW YORK.

The author declared that displacements of the uterus are not accorded the consideration they deserve, and that the routine plan of inserting a pessary and dismissing the case from further attention is an error too often committed. He thinks the majority of cases, especially those of long standing, where structural changes have taken place in the walls of the organ, require surgical intervention for their cure. The pessary alone is never sufficient, except, perhaps, in very recent cases. The concomitant metritis and endometritis must be overcome before a radical cure can be effected.

After discussing the merits of Alexander's operation and the intraperitoneal methods of shortening the round ligaments and vaginal fixations, he described an operation for retroflexion which he had employed with success for the past twelve years.

The Alexander operation, which is only applicable in movable retro-deviations, he thinks unnecessary. Its chief disadvantage is the time it requires and the prolonged convalescence it entails.

Intraperitoneal shortening of the round ligaments requires more

*Abstract of paper read before New York State Medical Society, January 28, 1896.

time for its execution, and the convalescence is longer than suspensio-uteri, and the results have not been so good.

Vaginal fixation is objectionable because it substitutes a fixed anteflexion for a movable posterior displacement. The recent unfavorable reports of complications during labor following it, offer another very serious objection to this operation. The best evidence of its inefficiency is that its originator, Mackinrodt, has abandoned it.

Where the uterus is fixed by firm adhesions, the author advocates opening the abdomen by means of a small incision, breaking them up and suspending the uterus by its posterior face from the anterior abdominal wall. This does not fix the organ as when ventro-fixation is done. In time the uterus recedes from the abdominal wall, close to which it is at first suspended, and swings in an easy position of nearly normal anteflexion. This he prefers to ventro-fixation, because the uterus occupies a nearly normal position and is fairly movable. Its execution consumes less time than intraperitoneal shortening of the round ligaments. The results have been very gratifying.

When the adhesions are not very firm or extensive, they are broken up by manipulations under anesthesia without opening the peritoneal cavity, and the case is then treated in the same manner as when the organ is movable.

The method of procedure which he advocates in place of Alexander's operation in movable retro-deviations, has this to recommend it, viz: that it aims at a cure of the coexisting metritis and endometritis, the maintaining cause of the displacement, and requires but a week's confinement in bed.

For retroversion he dilates the canal, curettes and packs the cavity with iodoform gauze. The vagina is then tamponed with the same gauze in such manner as to throw the uterus into a position of anteversion. This dressing is removed every day, the cavity is washed out with a one per cent. solution of lysol, and it is reapplied. This is done for a week, during which time the patient is confined to bed. Then a vaginal pessary is fixed to hold the uterus in a correct position. The cavity is irrigated twice a week until a healthy endometrium is reproduced.

For retroflexion the same procedure is adopted, but instead of packing the uterus with gauze, a straight glass drainage stem is used, which serves the purpose of a splint and keeps the uterus straight. It is then maintained in a position of anteversion by means of vaginal tampons of iodoform gauze. The gauze tampon and stem are removed every day, the cavity is irrigated to remove retained clots and débris, and after cleansing the stem it is re-inserted. At the end of a week the stem is removed, a vaginal pessary is inserted, and the patient is permitted to get up.

The success which he has obtained with this method leads him to believe that the other more complicated and hazardous operations designed for movable retro-deviations are unnecessary.

